# Minority Stressors, Rumination, and Psychological Distress in Lesbian, Gay, and Bisexual Individuals

**DOI:** 10.1007/s10508-019-01502-2

**Published:** 2019-07-22

**Authors:** Liadh Timmins, Katharine A. Rimes, Qazi Rahman

**Affiliations:** 1 Department of Psychology, Institute of Psychiatry, King’s College London, 5th Floor, Bermondsey Wing, Guy’s Hospital Campus, London, SE1 9RT UK; 2 Department of Psychology, Institute of Psychiatry, King’s College London, Denmark Hill, London, UK

**Keywords:** Minority stress, Gender nonconformity, Rumination, Prejudice, Sexual orientation

## Abstract

This study tested the mechanisms by which social stigma contributes to psychological distress in lesbian, gay, and bisexual individuals. A large community sample (*N* = 4248, *M* age = 29.9 years, 42.9% female, 57.1% male, 35.7% bisexual, 64.3% lesbian/gay, 9.9% non-white) was recruited using targeted and general advertisements for an online cross-sectional survey. Participants completed measures of childhood gender nonconformity, prejudice events, victimization, microaggressions, sexual orientation concealment, sexual orientation disclosure, expectations of rejection, self-stigma, rumination, and distress. Structural equation modeling was used to test the relationships between these variables in a model based upon minority stress theory and the integrative mediation framework with childhood gender nonconformity as the initial independent variable and distress (depression, anxiety, and well-being) as the final dependent variable. The results broadly support the hypothesized model. The final model had good fit *χ*^2^(37) = 440.99, *p* < .001, TLI = .96, CFI = .98, RMSEA = .05 [.05, .06] and explained 50.2% of the variance in psychological distress and 24.8% in rumination. Sexual orientation and gender had moderating effects on some individual paths. Results should be considered in the context of the cross-sectional nature of the data, which prevented tests of causality, and self-report measures used, which are vulnerable to bias. Findings indicate strong relationships between minority stressors and psychological distress in lesbian, gay, and bisexual individuals, which are partially accounted for by rumination. These results may inform the development of interventions that address the added burden of minority stress among lesbian, gay, and bisexual individuals.

**Electronic supplementary material:**

The online version of this article (10.1007/s10508-019-01502-2) contains supplementary material, which is available to authorized users.

## Introduction

Compared to heterosexual individuals, lesbian, gay, and bisexual (LGB) individuals are at substantially greater risk of a range of common mental disorders (King et al., 2008; Plöderl & Tremblay, 2015; Ross et al., 2018; Semlyen, King, Varney, & Hagger-Johnson, 2016). For example, the risks of anxiety and depression in LGB individuals are over 1.5 times higher than they are for heterosexual individuals, both in a period of 12 months or across the lifetime (King et al., 2008). As LGB individuals make up approximately 3.5% of the population (Gates, 2011), this constitutes a significant public health burden.

The most commonly cited cause of these disparities is “minority stress,” defined as the unique stress experienced by sexual minorities living in a social environment characterized by anti-LGB, or “heterosexist,” prejudice and stigma (Meyer, 2003). Minority stress theory includes four kinds of “stressors” unique to LGB individuals, which are broadly divided into “distal” and “proximal” stressors. Distal stressors are external events and conditions, such as victimization and discrimination, as well those of a lower intensity and more subtle nature, known as “microaggressions” (Nadal, Whitman, Davis, Erazo, & Davidoff, 2016). Proximal stressors constitute the minority individual’s cognitive processes, self-concepts, coping mechanisms, and other behaviors that contribute to their distress, including self-stigma, concealment, and expectations of rejection.^1^ “Self-stigma” refers to the process by which LGB individuals internalize heterosexist attitudes, “concealment” refers to the manner in which LGB individuals sometimes hide their minority status in order to avoid prejudice events, and “expectations of rejection” describes the way LGB individuals can come to expect prejudice events and become hypervigilant (Meyer, 2003).

**Fig. 1:**
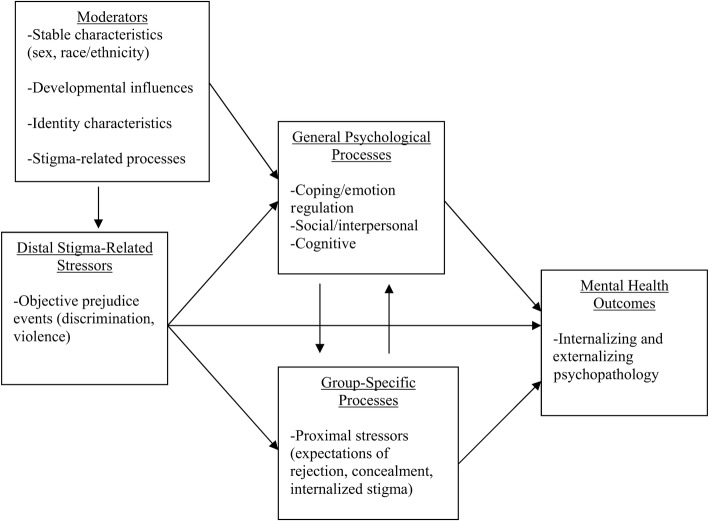
Hatzenbuehler’s integrative mediation framework of group-specific and general psychological processes. Reprinted from Hatzenbuehler (2009). Copyright 2009 by the American Psychological Association. Reprinted with permission

Expanding upon Meyer’s (2003) model, Hatzenbuehler (2009) hypothesized that stigma-related stress can result in higher levels of general, non-LGB-specific deleterious sequelae, such as rumination, which can further result in psychopathology. Furthermore, Hatzenbuehler (2009) hypothesized that proximal stressors may be mediators of the relationship between prejudice events and mental health outcomes. Finally, Hatzenbuehler (2009) noted that some of the paths between variables may be moderated by sex, race, developmental influences, identity characteristics, and stigma-related processes, and that these may affect levels of different stressors experienced. This integrative framework is illustrated in Fig. 1.

Meyer’s (2003) and Hatzenbuehler’s (2009) frameworks have inspired a large number of studies which attempt to determine the mechanisms by which minority stressors can cause and exacerbate mental health issues in LGB individuals. Meta-analyses have found that acute prejudice events, expectations of rejection, and internalized homophobia are all positively associated with distress in LGB individuals with small to medium effect sizes (Newcomb & Mustanski, 2010; Schmitt, Branscombe, Postmes, & Garcia, 2014), though research on concealment has been less straightforward (discussed further below). Furthermore, “brooding,” a subtype of rumination characterized by moody, passive contrasting of one’s responses to distress with an ideal, has been found to be associated with distress in convenience samples of LGB individuals (Hatzenbuehler, Dovidio, Nolen-Hoeksema, & Phills, 2009a; Lewis, Milletich, Mason, & Derlega, 2014; Szymanski, Dunn, & Ikizler, 2014). Research has also found that brooding rumination predicts later depression in LGB individuals (Hatzenbuehler, Nolen-Hoeksema, & Dovidio, 2009b). Support has also been found for a large number of indirect effects pertinent to Hatzenbuehler’s (2009) framework, which are outlined in Table 1. In summary, many specific paths and relationships predicted by these frameworks have been tested and found to indeed be significant.

**Table 1:** Studies finding significant indirect effects in line with Hatzenbuehler’s (2009) framework

References	A	B	C
Brewster et al. (2013)^a^	Biphobic prejudice events	Expectations of rejection	Psychological distress
Feinstein et al. (2012)	Heterosexist prejudice events	Internalized homophobia	Depression
	Heterosexist prejudice events	Internalized homophobia	Anxiety
	Heterosexist prejudice events	Gay-related rejection sensitivity	Depression
	Heterosexist prejudice events	Gay-related rejection sensitivity	Anxiety
Hatzenbuehler, Dovidio, et al. (2009)	Internalized homophobia	Brooding rumination	Psychological distress
Kaufman et al. (2017)	Microaggressions	Rumination	Depressive symptoms
Liao et al. (2015)	Expectations of rejection	Anger rumination	Psychological distress
Puckett et al. (2016)	Heterosexist victimization	Internalized homophobia	Internalizing symptoms
Velez et al. (2013)^b^	Prejudice events	Avoiding	Distress
Szymanski et al. (2014)	Internalized homophobia	Brooding rumination	Psychological distress via brooding rumination
Szymanski and Ikizler (2013)	Heterosexist prejudice events	Internalized heterosexism	Depression
	Heterosexist prejudice events	Internalized heterosexism	Social anxiety
	Heterosexist prejudice events	Gay-related rejection sensitivity	Depression
	Heterosexist prejudice events	Gay-related rejection sensitivity	Social anxiety

A = independent variable, B = intermediary variable, C = dependent variable, gay-related rejection sensitivity = anxious expectations of rejection based on one’s sexual minority status, avoiding = an identity management strategy and type of active concealment

^a^But not internalized biphobia or outness

^b^But not “counterfeiting” (presenting a false heterosexual identity)

Though this initial research is promising, none of these studies test a full model incorporating all four types of minority stressors and rumination. Additionally, though Meyer’s (2003) and Hatzenbuehler’s (2009) frameworks have been key to guiding and developing this research, an overreliance on the specific factors named in these frameworks may have limited studies on sexual minority mental health. For example, most extant research has treated concealment as monolithic and interchangeable with outness (disclosure of one’s sexual orientation to others). Despite this, several studies have found various conflicting findings on the relationship between outness and distress, which are outlined in Table 2.

**Table 2:** Findings on the relationship between outness and distress

Significant, positive	Nonsignificant	Significant, negative
Frost and Bastone (2008)	Brewster and Moradi (2010)	Cohen et al. (2016)
	Brewster et al. (2013)	Dyar et al. (2014)
	Cohen et al. (2016)	Frost et al. (2007)
	Fredriksen-Goldsen et al. (2013)	Lehavot and Simoni (2011)

These mixed results are perhaps unsurprising. Keeping one’s sexual orientation concealed may help LGB individuals avoid prejudice events in the short term, thus preventing some distress, but it may also be psychologically burdensome due to the constant threat of discovery and high demands required to successfully conceal (Pachankis, 2007). Indeed, most studies have found that prejudice events are actually positively associated with outness (Brewster & Moradi, 2010; Brewster, Moradi, DeBlaere, & Velez, 2013; Cook, Sandfort, Nel, & Rich, 2013; Lehavot & Simoni, 2011; cf. Kuyper & Fokkema, 2010). This is likely because individuals who are more out about their sexual orientation are more likely to experience prejudice events (Meyer, 2003; Pachankis, 2007).

Given these theoretical and empirical inconsistencies regarding the relationship between outness and distress, it makes little sense to conceptualize prejudice events as having indirect effects on distress via this aspect of concealment. Indeed, if outness is positively associated with both distress and prejudice events at some level, a more coherent hypothesis would be that outness causes more prejudice events, which cause higher levels of distress. At the same time, outness does appear to have ameliorative effects on self-stigma (Pistella, Salvati, Ioverno, Laghi, & Baiocco, 2016) and positive indirect effects of desire to keep sexual orientation undisclosed have been found on both depression and anxiety via internalized homophobia (Schrimshaw, Siegel, Downing, & Parsons, 2013). At first glance, this might seem contradictory, but these two paths are compatible with each other and the previous literature. There may simply be competing negative and positive indirect effects of outness on distress via other proximal stressors and prejudice events, respectively. While coming out might reduce proximal stress, in that it facilitates access to support, it may also increase exposure to distal stressors. In aggregate, these effects may be positive or negative depending on a litany of other variables. However, these explanation needs to be subject to good empirical testing.

In terms of implications for Hatzenbuehler’s (2009) framework, effects of outness on distress via other stressors would seem to suggest that outness should be repositioned. However, this does not mean that concealment should be moved in its entirety. In addition to not always disclosing their sexual orientation, LGB individuals also actively alter their behavior in order to avoid being identified as LGB and/or subjected to prejudice events (Button, 2004; D’Augelli, 1992; Kuyper & Fokkema, 2010; Pachankis & Goldfried, 2006). Though such altered behavior has been given a variety of names by different researchers, “active concealment” will be used here for simplicity. Indeed, active concealment of sexual minority status from coworkers has been found to be associated with distress in LGB individuals (Velez, Moradi, & Brewster, 2013). As such, some forms of concealment could still function in the manner suggested by Hatzenbuehler (2009) and indeed, along with other minority stressors and negative psychological processes, might form the mechanism by which outness is linked to distress. However, little other work examining this construct has been performed, making it difficult to reach a definitive conclusion.

A further issue with overreliance on these two major frameworks is that little attention is paid to the different experiences of different sexual minority subgroups, such as lesbian/gay and bisexual individuals. Though Meyer (2003) stated that bisexual individuals may be exposed to more stressors and have greater mental health problems than lesbian/gay individuals, none of the above-outlined studies or frameworks have considered sexual orientation subgroup as a potential moderator of pathways from minority stressors to their sequelae. Indeed, subsequent research has, for the most part, supported the idea that bisexual individuals experience worse mental health than lesbian/gay individuals (Plöderl & Tremblay, 2015; Ross et al., 2018). Additionally, bisexual individuals report experiencing unique prejudice events related to their bisexuality, which has implications for potentially different or higher self-stigma or other proximal stressors (Brewster & Moradi, 2010; Dyar, Feinstein, & London, 2014). As such, it is important that the specific ways in which minority stressors are linked to distress are tested.

Lower levels of outness are one stressor that may require particular attention when examining bisexual minority stress. Representative samples have found that bisexual individuals are substantially less likely to be out than lesbian/gay people (Herek, Norton, Allen, & Sims, 2010; Taylor, 2013). As such, if specific circumstances under which outness improves well-being can be identified, it may be an important target for intervention for this subgroup specifically. However, it is unlikely that bisexual individuals are less likely to be out without reason. Indeed, as bisexual individuals report experiencing prejudice events from both heterosexual and lesbian/gay individuals (Brewster & Moradi, 2010; Dyar et al., 2014) and it has been theorized that bisexual individuals who are not out may have an easier time blending into the general population than lesbian/gay individuals (Chung, 2001), there may simply be fewer benefits and more costs to being out as bisexual. Indeed, research has found that higher outness is associated with higher alcohol and drug abuse for bisexual women, but not for lesbians or queer women, corroborating this idea and offering a potential explanation for inconsistent findings about outness (Feinstein, Dyar, & London, 2017). In conclusion, sexual minority subgroup (being bisexual, gay, or another sexual minority) effects on the strength of the relationships between outness and other minority stressors should be assessed before bisexual individuals are encouraged to come out to alleviate their distress, and sexual minority subgroup should be conceptualized as a potential moderating factor in Hatzenbuehler’s (2009) framework, alongside sex and race.

A final factor that is neglected in both frameworks and much of the research based on them is gender nonconformity. Research has found that LGB men and women display higher levels of gender nonconformity than their heterosexual counterparts (Bailey & Zucker, 1995; Lippa, 2005, 2008). Gender nonconformity is also associated with higher levels of outness, more experiences of prejudice events, and more distress (Baams, Beek, Hille, Zevenbergen, & Bos, 2013; Cook et al., 2013; Feinstein, Goldfried, & Davila, 2012; Gordon & Meyer, 2008; Landolt, Bartholomew, Saffrey, Oram, & Perlman, 2004; Lehavot & Simoni, 2011; Rieger, Linsenmeier, Gygax, Garcia, & Bailey, 2010; Rieger & Savin-Williams, 2012; Roberts, Rosario, Slopen, Calzo, & Austin, 2013; Sandfort, Melendez, & Diaz, 2007). Indirect effects of gender nonconformity on distress have also been found via prejudice events in a variety of samples of LGB individuals (Baams et al., 2013; Sandfort et al., 2007; Van Beusekom, Bos, Kuyper, Overbeek, & Sandfort, 2016). Additionally, an indirect effect was found via internalized homophobia in one of these studies (Van Beusekom et al., 2016). As such, gender nonconformity may play a similar role to race and sex in Hatzenbuehler’s (2009) framework and affect levels of different stressors experienced and this should be tested.

Notably, nonsignificant relationships have been found between gender nonconformity and each of prejudice events, outness, and depression in sexual minority women alongside small, but significant positive relationships, depending on the measure used (Lehavot & Simoni, 2011). Additionally, the relationship between gender nonconformity and depression appears to be less pronounced in women in general than in men (Roberts et al., 2013). This perhaps reflects the fact that previous research has suggested that gender nonconformity is viewed as less socially acceptable in boys than in girls (Kane, 2006). At the same time, another study found that lesbian and bisexual women report experiencing more gender-nonconformity-related discrimination than gay and bisexual men (Gordon & Meyer, 2008). As such, there may be moderating effects of gender on these relationships and this should be examined in a sample large enough to detect small effects.

Finally, despite Hatzenbuehler’s (2009) proposition of sex as a moderating factor for the paths from minority stressors to distress, little research has actually tested this idea. This may be partly due to a lack of specific hypotheses. Some individual relationships that may be moderated by sex are those between minority stressors and both rumination and distress. Indeed, a large amount of research has found that the relationship between stressors and depression is stronger in women in general than in men (Tennant, 2002) and women have been found to respond to stressors with rumination more than men do (Garnefski, Teerds, Kraaij, Legerstee, & van Den Kommer, 2004). It is likely that this applies to sexual minority women as well.

**Fig. 2:**
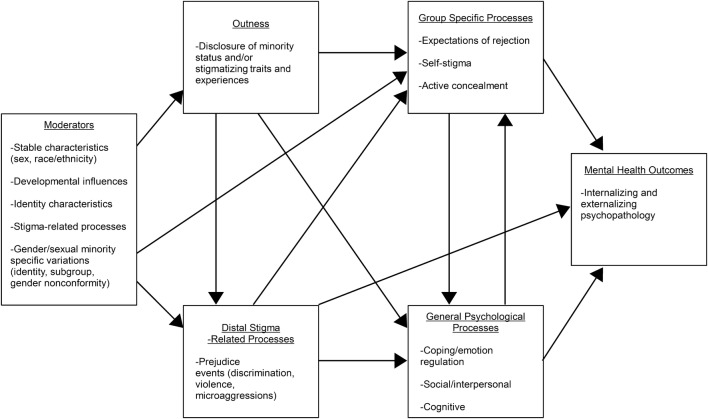
Modified integrative mediation framework. “Moderators” consist of variables that may moderate paths between other variables and/or affect levels of different stressors experienced, in line with Hatzenbuehler (2009)

In summary, a large amount of research based on Meyer’s (2003) and Hatzenbuehler’s (2009) frameworks has been performed, with promising results. However, in the frameworks and associated research a number of potential effects have been neglected, including sexual minority subgroup differences in the relationships between outness and other variables, effects of and on active concealment, indirect effects of outness via other stressors, and sex differences in the relationships between stressors and both rumination and distress. Reflecting these limitations and explanations, a modified version of Hatzenbuehler’s (2009) integrative mediation framework is proposed in Fig. 2.

### Present Study

**Fig. 3:**
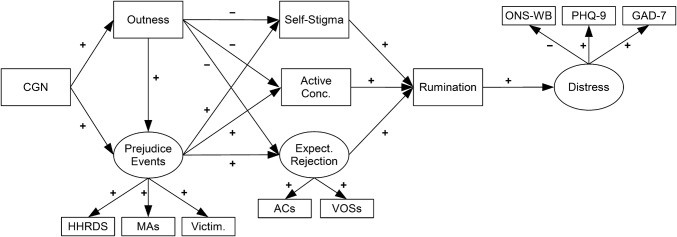
Hypothesized model. Paths predicted to be positive are marked with a (+), and paths predicted to be negative are marked with a (−). *HHRDS* heterosexist harassment, rejection, and discrimination scale, *MAs* microaggressions, *Victim.* victimization, *Active Conc.* active concealment, *Expect. Rejection* expectations of rejection, *ONS-WB* UK office for national statistics well-being measure, *ACs* acceptance concerns, *VOSs* vigilance for others’ suspicions scale, *PHQ-9* patient health questionnaire 9-item scale (depression), *GAD-7* generalized anxiety disorder 7-item scale

The present study had two major goals. Firstly, this study tested, for the first time, a new model of psychological distress in LGB individuals incorporating not only all four types of minority stressors from Meyer’s (2003) framework, but also rumination from Hatzenbuehler’s (2009) framework and gender nonconformity, which had not been included in either of these previous approaches. Based on the modified integrative mediation framework (see Fig. 2), the model also separated “outness” from “active concealment,” unlike much previous research. The new model is shown in Fig. 3. Secondly, this study tested a number of theoretically relevant gender and sexual orientation moderation effects based on the above-outlined literature and in line with the broad predictions of the modified integrative mediation framework. Predictions tested in this model were as follows:

#### **Hypothesis 1**

There would be an indirect effect of CGN on distress via higher levels of outness.

#### **Hypothesis 2**

Childhood gender nonconformity (CGN) and psychological distress would be positively associated, which would be explained by an indirect effect via greater experience of prejudice events.

#### **Hypothesis 3**

There would be a positive indirect effect of outness on distress via exposure to more prejudice events.

#### **Hypothesis 4**

There would be a negative relationship between outness and distress, which would be explained by negative indirect effects via lower levels of self-stigma, active concealment, and expectations of rejection.

#### **Hypothesis 5**

There would be a positive relationship between prejudice events and distress, which would be explained by positive indirect effects via active concealment, expectations of rejection, and self-stigma, in line with Hatzenbuehler (2009).

#### **Hypothesis 6**

There would be positive relationships between each of these three proximal stressors and distress, which would be explained by indirect effects via rumination (Hatzenbuehler, 2009).

#### **Hypothesis 7**

The relationship between outness and prejudice events would be stronger for bisexual individuals than for lesbian/gay individuals.

#### **Hypothesis 8**

The relationships between outness and each other proximal stressor would be stronger for lesbian/gay individuals than for bisexual individuals. This and hypothesis 7 were postulated due to the above-mentioned disparities in outness.

#### **Hypothesis 9**

All paths to rumination and distress would be stronger for women than for men (Garnefski et al., 2004; Hatzenbuehler, 2009; Tennant, 2002).

#### **Hypothesis 10**

The paths from CGN to both prejudice events and outness would be stronger for men than for women (Kane, 2006).

## Method

### Participants

A total of 7141 individuals completed the survey. Individuals who appeared to be giving “prank” answers (identified by antagonistic responses in open fields) were excluded from analysis (*n* = 9). In order to isolate issues experienced by LGB individuals, participants with a discordant gender identity and sex assigned at birth (i.e., transgender individuals; *n* = 1232), a heterosexual identity (*n* = 1021), any sexual orientation identity other than LGB (*n* = 519), or missing or unclear data for sexual orientation, gender identity or sex assigned at birth (*n* = 112) were also excluded. Though many transgender individuals identified as LGB, such individuals are subject to minority stressors relating to their gender identity that could be tapped into by the measures used in this study (Hendricks & Testa, 2012). Thus, these groups were analyzed separately (Timmins, Rimes, & Rahman, 2017). Non-LGB sexual minority groups, including asexual (*n* = 98), pansexual (*n* = 160), and queer (*n* = 80) individuals, were considered for inclusion in these analyses; however, subsamples of theses sizes are insufficient to reliably test for invariance across groups (Kline, 2011).

Sexual orientation was measured using four items. Firstly, participants were asked to indicate how they identify their sexual orientation using a multiple-choice question with the response options of “heterosexual (straight),” “bisexual,” “homosexual (gay/lesbian),” “asexual,” and “other sexual orientation (please specify).” This final response option had an open field wherein participants could write their own identity. Additionally, participants reported the relative frequency with which they experience sexual attraction, romantic attachments, and romantic infatuations for males and females with three respective items. Ratings were given on a 7-point scale, 0 = “always male,” 3 = “equally male and female,” 6 = “always female” with an eighth option for “little or no [sexual attraction].” Ratings were reverse-scored for women, and “little or no” responses were treated as missing data. Polychoric correlations indicated that for LGB-identified individuals sexual identity correlated very highly with sexual attraction (*r*[4212] = .95, lesbian/gay *M* = 5.66, SD = .59, bisexual *M* = 3.03, SD = 1.17), romantic attachments (*r*[4199] = .95, lesbian/gay *M* = 5.69, SD = .65, bisexual *M* = 2.56, SD = 1.55), and romantic infatuations (*r*[4197] = .95, lesbian/gay *M* = 5.77, SD = .54, bisexual *M* = 2.88, SD = 1.55). As there was little or no divergence between these four different dimensions, identity was solely used to separate bisexual and lesbian/gay individuals without further stratification in line with prior studies. This left a final total of 4248 LGB participants. Of these, 1828 were gay men, 902 were lesbian/gay women, 596 were bisexual men, and 922 were bisexual women. Further details about participant demographics are given in Table 3.

**Table 3:** Demographic information of participants

Sexual orientation	
Bisexual	35.7%
Gay	64.3%
Gender	
Woman	42.9%
Man	57.1%
Age	
Mean	29.9
Range	16–82
Country of residence	
UK	49.7%
USA	27.3%
Ireland	5.8%
Canada	3.9%
Australia	3.4%
Other—Western	8.4%
Other—non-Western	1.5%
Relationship status—%	
Single	44.3%
Living together	17.9%
Steady	14.4%
Married	11.6%
Casual	4.1%
Separated/divorced	1.5%
Other relationship status	6.3%
Race/ethnicity—%	
White	89.1%
Mixed race/ethnicity	3.6%
Asian	2.9%
Latino/Hispanic	2.3%
Black	1.2%
Other race/ethnicity	.8%

### Procedure

Ethical approval for the study was obtained from the university research ethics committee. Data were collected by means of an online survey in order to ensure anonymity of participants. Participants were recruited via targeted and general advertisements on online lesbian, gay, bisexual, transgender, and other gender and sexual minority (LGBT+) press websites, Internet forums, listservs, mailings lists, and social media sites. Participants were required to be aged 16 years or older. In order to facilitate the collection of a large sample, no geographical requirements for participation were imposed. Additionally, as these data were collected as part of a larger project, participants were invited to participate regardless of sexual orientation. Advertisements and posts included a link that when clicked on presented participants with the information sheet and consent form for the study.

### Measures

All measures were in English. Questionnaires covered demographic variables, CGN, sexual orientation, prejudice events, active concealment, outness, expectations of rejection, self-stigma, rumination, and psychological distress. Where necessary, measures were modified to be applicable to heterosexual, asexual, and transgender people.^2^ New measures were developed when appropriate measures could not be found (see below). For each new measure, novel items were generated based on research and theory and correlation matrices were visually inspected for collinear items (*r* > .90) and overall intercorrelations between item pairs. Parallel analyses were then performed for each set of items in order to determine the likely number of factors for each new scale (Horn, 1965). Parallel analysis represents a method for determining the number of factors to retain that is both objective and highly accurate (Hayton, Allen, & Scarpello, 2004; Velicer, Eaton, & Fava, 2000). Exploratory factor analyses were performed for each set of items using principal axis factor extraction and Geomin rotation (Yates, 1987). These procedures are in line with general recommendations for exploratory factor analysis (Russell, 2002). Factor loadings less than .32 were considered to be insufficiently strong to be of relevance as per Tabachnick and Fidell (2013). Cronbach’s alphas were calculated to determine internal reliability, and scales were considered acceptable if *α* > .70, good if *α* > .80, and excellent if *α* > .90 (Darren & Mallery, 2003). These analyses were performed using R-Menu, version 2.4 (Basto, 2015). Scale scores were then computed for each of the new measures, and correlation coefficients for theoretically relevant variables were calculated to determine whether the new scales displayed sufficient convergent and divergent validity.

Bivariate correlations with other study variables were inspected to ensure discriminant and convergent validity. Cronbach’s alphas, reported below, were calculated, and all measures displayed good internal reliability (*α* > .80). These measures can be seen in online Supplementary Tables S1–S3.

#### Demographic Variables

Data were taken on age, relationship status, race/ethnicity, country of residence, gender identity, and sex assigned at birth.

#### Childhood Gender Nonconformity

CGN was measured using the 10-item recalled childhood gender nonconformity scale (Hassan & Rahman, 2007). Participants indicated their levels of CGN from as early as they can remember to 12 years old on 10 items rated on various 5-point scales ranging from 1 to 5. Higher averaged scores reflected more gender nonconforming childhood behavior and interests. Example items include “as a child, my favourite toys and games were:” with response ranging from 1 = “always ‘boy-like’” to 5 = “always ‘girl-like’” and “in fantasy or pretend play, I took the role of:” with response ranging from 1 = “only of boys or men” to 5 = “only of girls or women.” This measure has displayed excellent internal consistency in a sample of gay men and is derived from a longer version which displayed good internal consistency in a sample of lesbian/gay individuals and validity in the form of large differences between sexual orientation groups (Hassan & Rahman, 2007; Zucker et al., 2006). Cronbach’s *α* = .85 in the present study.

#### Disclosure

Disclosure of sexual minority status was assessed using a version of an outness measure (Meyer, Rossano, Ellis, & Bradford, 2002). Participants indicated the proportion of people that they were “out to” about their sexual orientation among each of “family,” “LGBT+ friends,” “heterosexual friends,” “coworkers,” and “healthcare professionals” on a 4-point scale, ranging from 1 = “out to none” to 4 = “out to all.” Higher averaged scores indicated high levels of outness. A version of this scale displayed acceptable internal reliability and good validity in LGB individuals (Frost & Meyer, 2009). Cronbach’s *α* = .88 in the present study.

#### Prejudice Events

Experiences of harassment, rejection, and discrimination due to LGBT+ status were measured using a version of the heterosexist harassment, rejection, and discrimination scale (Szymanski, 2006), modified to be applicable to LGBT+ individuals as whole and those perceived as LGBT+, rather than just gay and lesbian individuals. Participants rated the frequency with which they had experienced 14 events in the past year because they are LGBT+ or were perceived to be on a 6-point scale ranging from 1 = “The event has never happened to you” to 6 = “The event happened almost all of the time (more than 70% of the time)” (*α* = .91). Example items include “How many times have you been called an offensive heterosexist/transphobic name, like faggot, tranny, dyke or other names?” and “How many times were you denied a raise, a promotion, tenure, a good assignment, a job, or other such thing at work that you deserved because you are LGBT+ or were perceived to be LGBT+?” Versions of this scale have displayed good validity and internal reliability for LGB men and women (Szymanski, 2006, 2009). Cronbach’s *α* = .91 in the present study.

Lifetime experiences of victimization were measured using a measure from D’Augelli (2006) adapted to be applicable to LGBT+ individuals as whole and those perceived as LGBT+, rather than just LGB individuals. Participants were asked to rate how often they had experienced seven forms of victimization because they are LGBT+ or were perceived to be on a 4-point scale ranging from 0 = “Never” to 3 = “Three or more times.” Events assessed in the victimization measure were more severe than those assessed in the HHRDS. Examples include “verbal abuse” and “objects being thrown.” Versions of this scale have shown good validity and internal reliability for sexual minority women and been used with sexual minority men, although psychometric data were not provided (D’Augelli, 2006; Lehavot & Simoni, 2011). Cronbach’s *α* = .84 in the present study.

Experiences of microaggressions in the past year were assessed using the sexual minority subscale of the gender and sexual minority microaggressions scale. This was developed for the current study based on theory and qualitative research on microaggressions experienced by LGB individuals (Nadal et al., 2016). Participants indicated how often in the past year they had experienced nine different microaggressions. Individual items were rated on a 5-point scale, ranging from 1 = “Never” to 5 = “All of the Time.” Example items include “people finding you fascinating or exotic because you are LGBT+ or they perceive you to be LGBT+” and “people accusing you of being defensive or sensitive when talking about your gender identity or sexual orientation.” Cronbach’s *α* = .85.

#### Active Concealment

Active concealment of LGB status was assessed using the gender and sexual minority presentation management inventory. This was developed for the current study based on theory and self-reported concealment strategies used by LGB individuals (D’Augelli, 1992; Pachankis & Goldfried, 2006). Participants indicated how often they engaged in five strategies in order to not appear LGBT+. Each item was rated on a 5-point scale, ranging from 1 = “Never” to 5 = “All of the Time.” Example items are “I try to act more masculine or feminine” and “I check myself to see if anything gives me away.” Cronbach’s *α* = .88.

#### Expectations of Rejection

Concerns with the potential of being stigmatized for being LGBT+ was measured using a version of the acceptance concerns subscale of the lesbian, gay, and bisexual identity scale (Mohr & Kendra, 2011), adapted to be applicable to LGBT+ individuals as a whole and those perceived as LGBT+, rather than just LGB individuals. These types of concerns are described as a form of expectations of rejection within Meyer’s (2003) framework. Participants rated on a 6-point scale ranging from 1 = “Disagree Strongly” to 6 = “Agree Strongly” three statements on their concerns over potentially being stigmatized for being LGBT+ or perceived as such. This scale has displayed acceptable internal reliability and construct validity with LGB individuals (Mohr & Kendra, 2011). Cronbach’s *α* = .87 in the present study.

Vigilance for others’ suspicions of own LGBT+ status and likely reactions were measured using the vigilance for others’ suspicions scale. This was developed for the current study based on theories of vigilance in general, concealable stigmas (Pachankis, 2007). Participants indicated on a 5-point scale ranging from 1 = “Never” to 5 = “All of the Time” how often they experienced three different forms of vigilance. Example items include “I pay close attention to whether people suspect me of being LGBT+” and “I am quick to notice changes in how someone is treating me if they have reason to suspect me of being LGBT+”; Cronbach’s *α* = .83.

#### Self-Stigma

Self-stigma was assessed using a version of the revised internalized homophobia scale, modified to be applicable regardless of gender identity (Herek, Gillis, & Cogan, 2009). Participants indicated on 5-point scale ranging from 1 = “Strongly Disagree” to 5 = “Strongly Agree” five statements about experiencing sexual orientation self-stigma. Example items include “I would like to get professional help in order to change my sexual orientation from what it is to something else” and “I feel that being of my sexual orientation is a personal shortcoming for me.” This measure has displayed good internal reliability and construct validity with LGB individuals (Herek et al., 2009). Cronbach’s *α* = .81 in the present study.

#### Rumination

Rumination was assessed using a version of the brooding subscale of the ruminative responses scale (Treynor, Gonzalez, & Nolen-Hoeksema, 2003), modified to refer to broad psychological distress rather than just depression and negative mood. Participants indicated on a 4-point scale ranging from 1 = “Almost never” to 4 = “Almost always” how frequently they experience five different cognitions when they feel down, sad, or distressed. Example items include “think about a recent situation, wishing it had gone better” and “think ‘Why do I always react this way?’” This measure has displayed good internal reliability in LGB individuals (Hatzenbuehler et al., 2009a, 2009b). The measure is also associated with both concurrent and long-term depression in the general population (Treynor et al., 2003). Cronbach’s *α* = .83 in the present study.

#### Psychological Distress

Depression was assessed using the patient health questionnaire 9-item scale (Kroenke, Spitzer, & Williams, 2001). Participants indicated on a 4-point scale ranging from 0 = “Not at all” to 3 = “Nearly every day” the frequency with which they had experienced nine different symptoms of depression over the previous 2 weeks. Example items include “little interest or pleasure in doing things” and “feeling down, depressed or hopeless.” It has high internal reliability with LGB individuals, demonstrates good construct validity in the general population, and is used in both research and clinical settings (Cochran, Balsam, Flentje, Malte, & Simpson, 2013; Martin, Rief, Klaiberg, & Braehler, 2006). Cronbach’s *α* = .92 in the present study.

Anxiety was assessed using the generalized anxiety disorder 7-item scale (Spitzer, Kroenke, Williams, & Löwe, 2006). Participants indicated on a 4-point scale ranging from 0 = “Not at all” to 3 = “Nearly every day” the frequency with which they had experienced seven different symptoms of anxiety over the previous 2 weeks. Example items include “feeling nervous, anxious or on edge” and “not being able to stop or control worrying.” It has displayed high internal reliability with LGB individuals, demonstrates good construct validity in the general population, and is used in both research and clinical settings (Lehavot & Simoni, 2011; Löwe et al., 2008; Woodford, Han, Craig, Lim, & Matney, 2014). Cronbach’s *α* = .92 in the present study.

Well-being was assessed using the UK office of national statistics well-being measure (Self, Thomas, & Randall, 2012). Participants rated four aspects of their well-being on an 11-point scale ranging from 0 = “not at all” to 10 = “completely.” Example items include “overall, how satisfied are you with your life nowadays?” and “overall, how happy did you feel yesterday?” The items from this scale have been used in annual, nationally representative surveys in the UK since 2011. Cronbach’s *α* = .82 in the present study.

### Data Analysis

#### Missing Data

Participants were not required to respond to any questions, in case answering caused discomfort or the item was not applicable. Missing data ranged from 0.2 to 2.2% on study variables, meaning that similar results could be expected across missing data procedures (Tabachnick & Fidell, 2013). Individuals’ scores for scales with missing items were calculated by substituting the mean of their remaining items, but only if 80% or more were complete, as this technique has been found to be robust at this level of item missingness (Roth, Switzer, & Switzer, 1999). This ensured scores were based on individuals’ own responses where this was possible without substantially affecting reliability. Full maximum likelihood estimation was used in main analyses for outstanding missing data (Enders & Bandalos, 2001). Pairwise deletion was used in preliminary analyses as full maximum likelihood estimation was not available for these tests in SPSS. Across preliminary analyses, 0.0–1.5% of cases were excluded.

#### Main Analysis

The hypothesized models were tested using structural equation modeling in AMOS, version 21.0 (Arbuckle, 2012). Strict definitions of mediation require tests of temporal precedence and maintain that cross-sectional data can only test for indirect effects (Kline, 2015). As such, we tested for total associations between variables and whether such relationships could be accounted for by indirect effects through intermediary variables, rather than mediation per se. To determine the degree to which the data fit the measurement model, a confirmatory factor analysis was performed with all variables in Fig. 3 included. Error terms for manifest variables loading onto the same factor were allowed to correlate if there was both theoretical justification and significant improvement of model fit. All possible cross-loadings of manifest variables were directly tested in their own alternative model.

The path from CGN to psychological distress was initially tested. Independent variables were then added to the model one at a time moving from earlier to later independent variable in the sequence outlined in the theoretical model. This allows for a complete model based on the hypotheses to be tested when the final variable was added, while also allowing initial relationships between independent variables and distress and whether those associations could be explained by indirect effects to be tested. As such, outness was next added to the model and the indirect path from CGN to psychological distress via outness was tested. Prejudice events were then added to the model, and the indirect paths from outness and CGN though prejudice events were tested. Expectations of rejection, active concealment, and self-stigma were then each separately added to the model, and the indirect effects of prejudice events, outness, and CGN on psychological distress via these variables were tested. These intermediary variables were then tested simultaneously in the same manner with their error terms allowed to correlate. Rumination then was added to the model, and the indirect effects of all other variables on psychological distress via rumination were tested.

Finally, the model was tested controlling for age, sex (0 = male; 1 = female), race/ethnicity (0 = white; 1 = non-white), sexual orientation (0 = lesbian/gay; 1 = bisexual), country of residence (four indicator variables with “UK” as the comparison consisting of “U.S./Canada,” “Australia/New Zealand,” “Other European Economic Area/Switzerland,” and “Other”), and relationship status (two indicator variables with “single” as the comparison consisting of “partnered” and “other,” such as separated or in a casual relationship).

In each model, paths that were nonsignificant were deleted if doing so did not significantly weaken model fit. Significance of indirect effects was tested using Sobel tests (Sobel, 1982). Though bootstrapping methodologies are often considered the “gold-standard” test of the significance of indirect effects due to their high levels of power, their Type I error rate is high (Fritz, Taylor, & MacKinnon, 2012). Thus, it is recommended that a test is chosen a priori based on whether avoiding Type I or Type II error is of greater concern and that the significances of the individual paths to and from the intermediary variable are also examined. Given the sample was far larger than the minimum required to achieve .80 power in a variety of appropriate tests, it was determined that the Sobel test should be chosen because of, not despite, its extremely conservative nature (Fritz & MacKinnon, 2007).

In line with general recommendations (Jackson, Gillaspy, & Purc-Stephenson, 2009), the chi-square test, an index to describe incremental fit (comparative fit index [CFI] and Tucker–Lewis index [TLI]) and a residuals-based measure (root-mean-square error of approximation [RMSEA]) were recorded. Good model fit was considered to be a CFI and TLI ≥ .95 and an RMSEA ≤ .60 (Hu & Bentler, 1999). Significant changes in model fit were tested for nested models using chi-square difference tests, and the better-fitting model was considered to be the model with the lowest Akaike information criterion (AIC; Akaike, 1987).

When the final model was selected, moderation analyses were performed to determine whether there were moderating effects of gender on all paths to rumination, all paths to psychological distress, and the path from CGN to outness, and sexual orientation (bisexual vs. lesbian/gay) moderation effects on all paths from outness to other minority stressors. All paths were left free to vary across groups, and each of the test paths was constrained to be equal across groups one at a time. Moderation was considered to have occurred if a constraint significantly worsened model fit according to a chi-square difference test (Ryu & Cheong, 2017). Finally, so that new hypotheses can be generated for future research, exploratory post hoc moderation analyses comparing bisexual men, bisexual women, gay men, and lesbians on all paths were performed. The results of these can be seen in online Supplementary Tables S4–S16.

## Results

### Preliminary Analysis

Descriptive statistics and bivariate correlations are given in Table 4. Bonferroni adjusted *t*-tests were performed in order to determine whether bisexual individuals differed from lesbian/gay individuals in anxiety, depression, well-being, and/or outness and whether women differed from men in anxiety, depression, well-being, and/or rumination (*α* = .05/8 = .006). Women had significantly higher levels of depression (female *M* = 10.34, SD = 7.13; male *M* = 9.54, SD = 7.13), anxiety (female *M* = 9.22, SD = 5.97; male *M* = 8.31, SD = 6.06), and rumination (female *M* = 2.58, SD = .74; male *M* = 2.43, SD = .77) than men, and significantly lower levels of outness were found for bisexual individuals (*M* = 2.37, SD = .82) relative to lesbian/gay (*M* = 3.31, SD = .75) individuals (all *p*s < .001). Differences in mental health between bisexual and lesbian/gay individuals were nonsignificant.

**Table 4:** Bivariate correlations and descriptive statistics for manifest variables

Variable	1	2	3	4	5	6	7	8	9	10	11	12	13
1. CGN	–												
2. Out	.14***	–											
3. Vict	.17***	.36***	–										
4. HRD	.21***	.18***	.57***	–									
5. MAs	.20***	.21***	.45***	.62***	–								
6. Conc	− .14***	− .26***	.18***	.33***	.34***	–							
7. SS	.04*	− .27***	.00	.14***	.14***	.50***	–						
8. ACs	− .11***	− .12***	.17***	.34***	.40***	.54***	.41***	–					
9. VOSs	− .08***	− .20***	.14***	.31***	.36***	.65***	.42***	.67***	–				
10. Rum	.09***	− .11***	.16***	.28***	.30***	.36***	.27***	.41***	.34***	–			
11. WB	.10***	.12***	− .19***	− .28***	− .21***	− .34***	− .28***	− .32***	− .28***	− .54***	–		
12. Anx	− .07***	− .06***	.23***	.35***	.33***	.35***	.24***	.36***	− .31***	.61***	− .68***	–	
13. Dep	.09***	− .07***	.25***	.35***	.30***	.35***	.27***	.33***	.30***	.59***	− .76***	.79***	–
*M*	2.77	2.97	.79	1.73	2.44	2.00	1.83	3.38	2.22	2.49	5.87	8.70	9.88
*SD*	.69	.90	.73	.74	.82	.77	.89	1.40	1.02	.76	2.05	6.04	7.19
Range	1–5	1–4	0–3	1–6	1–5	1–5	1–5	1–6	1–5	1–4	0–10	0–21	0–27

*CGN* childhood gender nonconformity, *Out* outness, *Vict* victimization, *HHRDS* heterosexist harassment, rejection, and discrimination scale, *MAs* sexual minority microaggressions, *Conc* gender and sexual minority presentation management inventory, *SS* self-stigma, *ACs* acceptance concerns, *VOSs* vigilance for others’ suspicions scale, *Rum* rumination, *WB* well-being, *Anx* anxiety, *Dep* depression

*p < .05; ***p < .001

### Measurement Model

The measurement model had a close-to-acceptable fit, *χ*^2^(42) = 996.85, *p* < .001, TLI = .91, CFI = .96, RMSEA = .07 [.07, .08], AIC = 1120.85; however, victimization could be allowed to negatively load onto the latent variable of expectations of rejection (*β* = − .29, *p* < .001). Notably, two of the victimization scale’s seven items ask about threats (“threats of physical attack” and “threats with weapons”) and one item refers to “being followed.” It is possible that participants interpreted the two threat items as referring to the general possibility of assault, rather than specific statements of an intention to harm them. Additionally, noticing or thinking that one is being followed may require a high amount of vigilance for prejudice events. This is in contrast to the HHRDS, which only mentions threats in one item and does this as part of a list of concrete behaviors (“made fun of, picked on, pushed, shoved, hit, or threatened with harm”) and the microaggressions measure, which asks about specific interactions in all its items. Thus, the victimization measure may not be cleanly tapping into its target construct, at least compared to the two other measures of prejudice events, which did not load onto expectations of rejection.

Given this, victimization was removed to ensure only specific measures of prejudice events were used. This resulted in good model fit: *χ*^2^(31) = 466.18, *p* < .001, TLI = .95, CFI = .98, RMSEA = .06 [.05, .06], AIC = 584.18. Allowing error terms for well-being and depression to correlate would have significantly improved model fit, as would have allowing error terms for well-being and anxiety. As there was no theoretical reason to pick one of these over the other and good model fit had already been achieved, these were not allowed to correlate. Factor loadings for the indicators of each latent variable in this model were quite high (*β* > .70).

### Transitional Models

A weak positive relationship was found between CGN and distress in the initial model (*β* = .10, *p* < .001). Adding outness to the model slightly increased this effect (*β* = .11, *p* < .001), and the paths from CGN to outness (*β* = .14, *p* < .001) and from outness to psychological distress were both significant (*β* = − .10, *p* < .001), which made for a significant negative indirect effect (*β* = − .01, *p* < .001), as predicted by Hypothesis 1. Prejudice events were then added to the model. This caused the relationship between CGN and distress to become nonsignificant (*β* = .00, *p* = .935). Retaining this path did not improve model fit (*χ*^2^[1] = .01, *p* = .935), and so it was deleted. Additionally, the paths from CGN to prejudice events (*β* = .23, *p* < .001) and prejudice events to distress were significant (*β* = .50, *p* < .001), which made for a significant indirect effect (*β* = .12, *p* < .001), as predicted by Hypothesis 2. The path from outness to prejudice events was also significant (*β* = .21, *p* < .001), which made for a significant indirect effect of outness on distress via prejudice events (*β* = .11, *p* < .001), as predicted by Hypothesis 3. This addition also caused the strength of the relationship between outness and distress to increase (*β* = − .21, *p* < .001).

When active concealment, expectations of rejection, and self-stigma were added to the model, a similar pattern was observed for each. The paths from outness and prejudice events to distress decreased but remained significant, as predicted by Hypotheses 4 and 5. A significant positive path was found from each newly added variable to distress, a significant negative path was found from outness to each newly added variable, a significant positive path was found from prejudice events to each newly added variable, and significant indirect effects of outness and prejudice events via each newly added variable were found, as predicted by Hypotheses 4 and 5 (see Table 5). This remained true for expectations of rejection and self-stigma when all three variables were all added at once, but the path from active concealment to distress became nonsignificant (*β* = .03, *p* = .094). Retaining this path did not improve model fit (*χ*^2^[1] = 2.79, *p* = .094). As active concealment was no longer associated with distress, it was removed from the model (see Table 5). This partly conflicts with predictions by Hypotheses 4 and 5. Through this process, all models remained within the above-mentioned standards for good model fit.

**Table 5:** Indirect effects on psychological distress

Indirect path	A	B	C	Indirect
Single-mediator models				
Prejudice events via active concealment	.47***	.12***	.47***	.06***
Outness via active concealment	− .22***		− .18***	− .03***
Prejudice events via self-stigma	.26***	.17***	.46***	.04***
Outness via self-stigma	− .33***		− .15***	− .06***
Prejudice events via ERs	.64***	.21***	.36***	.14***
Outness via ERs	− .35***		− .13***	− .07***
Multiple-mediator model				
Prejudice events via self-stigma	.26***	.12***	.37***	.03***
Outness via self-stigma	− .33***		− .12***	− .04***
Prejudice events via ERs	.64***	.15***		.10***
Outness via ERs	− .36***			− .05***
Full model, controlling for sexual orientation				
Self-stigma via rumination	.07***	.55***	.09***	.04***
ERs via rumination	.33***		–	.18***
Prejudice events via rumination	.19***		.24***	.10***

A = path from independent variable to intermediary variable, B = path from intermediary variable to distress, C = remaining direct path from independent variable to distress, Indirect = indirect path from independent variable to distress. Blank cells are redundant. Dashed cells represent deleted paths

***p < .001

### Final Structural Model

**Fig. 4:**
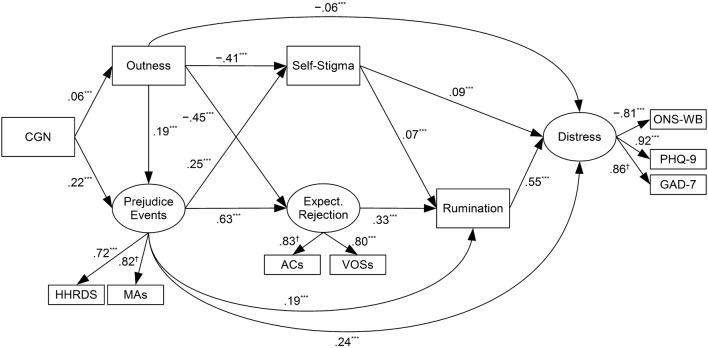
Final model. Sexual orientation was included as a control variable in model, but is omitted from figure for coherence, as is the case for error terms and correlation between error terms of self-stigma and expectations of rejection. *HHRDS* heterosexist harassment, rejection, and discrimination scale, *MAs* microaggressions, *Active Conc.* active concealment, *Expect. Rejection* expectations of rejection, *ACs* acceptance concerns, *VOSs* vigilance for others’ suspicions scale, *ONS-WB* ONS well-being measure, *PHQ-9* patient health questionnaire 9-item scale (depression), *GAD-7* generalized anxiety disorder 7-item scale. ***p < .001; ^†^*p* not calculated as path constrained to be equal to 1

Rumination was added to the model, and all paths were found to be significant (*p*s < .001) with the exception of the path from expectations of rejection to psychological distress (*β* = .00, *p* = .879). This path was deleted as it did not significantly affect model fit (*χ*^2^[1] = .23, *p* = .880). This model had good fit. *χ*^2^(32) = 365.26, *p* < .001, TLI = .96, CFI = .98, RMSEA = .05 [.05, .05]. Adding all control variables to the model caused the path from outness to rumination to become nonsignificant, and individual testing of control variables revealed that controlling for sexual orientation specifically caused this (*β* = − .04, *p* = .077). Deleting this path did not significantly affect model fit (*χ*^2^[1] = 3.21, *p* = .077). Otherwise, the overall pattern of results remained the same. As such, all control variables except for sexual orientation were left out of the final model. This model had good fit *χ*^2^(37) = 440.99, *p* < .001, TLI = .96, CFI = .98, RMSEA = .05 [.05, .06] and explained 50.2% of the variance in psychological distress and 24.8% of the variance in rumination (see Fig. 4). Significant indirect effects of prejudice events, expectations of rejection, and sexual orientation self-stigma on distress via rumination were found (see Table 5), as partly predicted by Hypothesis 6.

### Moderation

**Fig. 5:**
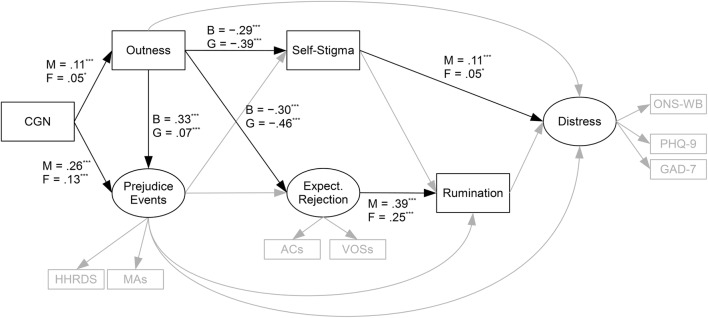
Moderated paths. *Active Conc.* active concealment, *Expect. Rejection* expectations of rejection, *B* score for bisexual group, *G* score for lesbian/gay group, *M* score for male group, *F* score for female group. *HHRDS* heterosexist harassment, rejection, and discrimination scale, *MAs* microaggressions, *Active Conc.* active concealment, *Expect. Rejection* expectations of rejection, *ACs* acceptance concerns, *VOSs* vigilance for others’ suspicions scale, *ONS-WB* ONS well-being measure, *PHQ-9* patient health questionnaire 9-item scale (depression), *GAD-7* generalized anxiety disorder 7-item scale. *p < .05; ***p < .001

As the sexual orientation variable was meaningless within each sexual orientation group, this was removed for the sexual orientation moderation analyses. Sexual orientation moderated the paths from outness to expectations of rejection (*χ*^2^[1] = 34.25, *p* < .001), from outness to self-stigma (*χ*^2^[1] = 22.08, *p* < .001), and from outness to prejudice events (*χ*^2^[1] = 56.75, *p* < .001). The paths from outness to both expectations of rejection and self-stigma were stronger for lesbian/gay individuals relative to bisexual individuals, whereas the path from outness to prejudice events was stronger for bisexual individuals relative to lesbian/gay individuals (see Fig. 5), as predicted by Hypotheses 7 and 8.

Sex did not moderate the paths from prejudice events to rumination (*χ*^2^[1] = .00, *p* = .991), from self-stigma to rumination (*χ*^2^[1] = .80 *p* = .370), from outness to distress (*χ*^2^[1] = 2.85, *p* = .091), from prejudice events to distress (*χ*^2^[1] = .190, *p* = .663), or from rumination to distress (*χ*^2^[1] = .21, *p* = .651), in conflict with Hypothesis 9’s predictions. Sex did, however, moderate the paths from expectations of rejection to rumination (*χ*^2^[1] = 9.43, *p* = .002), from self-stigma to distress (*χ*^2^[1] = 4.16, *p* = .041), from CGN to outness (*χ*^2^[1] = 9.00, *p* = .003), and from CGN to prejudice events (*χ*^2^[1] = 24.24, *p* < .001). Each of these paths was significant and positive for both groups, but stronger for men than for women (see Fig. 5). The former two paths were in the opposite direction predicted by Hypothesis 9, and the latter two paths were in line with Hypothesis 10’s predications.

## Discussion

This is the largest study investigating the relationships between multiple minority stressors and mental health in sexual minority individuals to date, as well as the only study testing a model incorporating all four types of minority stressors from Meyer’s (2003) minority stress theory alongside rumination, a clinically relevant predictor of psychological distress. All hypotheses were either fully or partially supported by the results of the current study, broadly supporting the new modified integrative mediation framework and corroborating a number of previously described existing findings and indirect effects outlined in Hatzenbuehler’s (2009) original framework (Hypotheses 5 and 6; Brewster et al., 2013; Feinstein et al., 2012; Hatzenbuehler, Dovidio, et al., 2009; Kaufman, Baams, & Dubas, 2017; Liao, Kashubeck-West, Weng, & Deitz, 2015; Puckett, Newcomb, Garofalo, & Mustanski, 2016; Szymanski et al., 2014; Szymanski & Ikizler, 2013; Velez et al., 2013).

As predicted, there were indirect effects of gender nonconformity on distress via outness and prejudice events (Hypotheses 1 and 2). These paths are not, of course, new. They corroborate previous evidence which links this trait to these minority stressors (Baams et al., 2013; Cook et al., 2013; Feinstein et al., 2012; Gordon & Meyer, 2008; Landolt et al., 2004; Lehavot & Simoni, 2011; Rieger et al., 2010; Rieger & Savin-Williams, 2012; Roberts et al., 2013; Sandfort et al., 2007). However, these results demonstrate that indirect effects of gender nonconformity on distress via prejudice events can account for the link between gender nonconformity and distress, and that the link between prejudice events and distress can be partially accounted for by an indirect effect via rumination. This underlines the importance of including gender nonconformity in Hatzenbuehler’s (2009) framework. Future research should examine the paths from this variable to distress longitudinally. Researchers may also wish to explore gender nonconformity as a moderator of some of the paths within the framework, as Hatzenbuehler conceived of several variables functioning as both (a) independent variables which affect the degree to which certain minority stressors are experienced and (b) moderators of the various paths from minority stressors to distress (see Figs. 2, 3).

As also predicted, there were concurrent indirect positive effect of outness on psychological distress via prejudice events and indirect negative effects via self-stigma and expectations of rejection (Hypotheses 3 and 4, respectively), suggesting that outness in the framework should be repositioned as per Fig. 2. Additionally, these competing positive and negative indirect effects may help explain the previously outlined mixed results for the relationship between outness and psychological distress (Brewster & Moradi, 2010; Brewster et al., 2013; Cohen, Blasey, Taylor, Weiss, & Newman, 2016; Dyar et al., 2014; Fredriksen-Goldsen et al., 2013; Frost & Bastone, 2008; Frost, Parsons, & Nanín, 2007; Lehavot & Simoni, 2011). These studies’ results may have differed from each other as their participants experienced differing amounts of increases in prejudice events and decreases in proximal stressors, possibly due to specific characteristics of the sample. Indeed, the present results have suggested that bisexuality may be one fact that affects changes in these variables after coming out (Hypotheses 7 and 8), and previous research has found that outness and health outcomes are differentially associated for bisexual individuals (Feinstein et al., 2017) and for men and women, depending on recency of coming out (Pachankis, Cochran, & Mays, 2015). Future research should conceive of prejudice events and proximal stressors as potential mediators between outness and distress, attempt to verify the direction of these relationships, and further examine the subgroups and circumstances that modulate the benefits and risks of coming out. Interested researchers may wish to use this paper’s exploratory analyses in Supplemental Materials to generate hypotheses for their future research. Additionally, clinicians should be wary of assuming that outness is necessarily beneficial in all cases, particularly when it comes to bisexual individuals.

Stronger relationships between outness and prejudice events for bisexual individuals and between outness and both self-stigma and expectations of rejection for lesbian/gay individuals were found, in line with Hypotheses 7 and 8, respectively. This suggests that sexual orientation subgroup should be included as a potential moderator of pathways from minority stressors to their sequelae in Hatzenbuehler’s (2009) framework. These results also dovetail with Feinstein et al.’s (2017) study, which found that outness is associated with negative outcomes (i.e., drug and alcohol abuse) for bisexual women, but not for lesbians or queer women. In addition, these results corroborate outness as a specific variable which seems to have less benefits for bisexual individuals than for lesbian/gay individuals. Future research should examine bisexuality as a variable that may moderate paths with Hatzenbuehler’s (2009) framework. As for clinicians, in addition to exercising caution with assuming that outness is beneficial for bisexual individuals, they should be advised that the results of clinical research that does not differentiate bisexual and lesbian/gay individuals should not be assumed to be applicable to the former of these two groups (e.g., Pachankis, Hatzenbuehler, Rendina, Safren, & Parsons, 2015).

Notably, the results also found that the paths from CGN to both outness and prejudice events were stronger for men relative to women, in line with Hypothesis 10, supporting the inclusion of sex as a moderating variable in the integrative mediation framework (Hatzenbuehler, 2009). Of particular note was the extremely weak relationship between gender nonconformity and outness in women, which would be difficult to detect in a sample smaller than that used here. This might explain the nonsignificant results for this relationship found by Lehavot and Simoni (2011). It is not necessarily the case that outness is not related to gender nonconformity; the relationship may just be hard to find because it is so small. Indeed, this relationship may have little importance for women as a result.

In conflict with predictions, however, were the rest of the study’s findings regarding sex as a moderator. All tests of sex’s moderating effect on paths to rumination and distress produced either nonsignificant results or significant results in the opposite direction to that proposed by Hypothesis 9. Specifically, the paths from expectations of rejection to rumination and from self-stigma to distress were stronger for men than for women. Given that, in general, women show greater responses to social rejection stressors and lower levels of self-esteem (Bleidorn et al., 2015) it is possible that this reflects the tendency of gay and bisexual men to resemble heterosexual women on sexually dimorphic psychological traits, such as levels of gender conformity and cognition, while lesbian and bisexual women tend to show opposite patterns (e.g., Rahman & Yusuf, 2015; Rieger, Linsenmeier, Gygax, & Bailey, 2008). Thus, the associations between minority stressors, rumination, and distress processes might also show this sex-inversed pattern (Savin-Williams, Cohen, Joyner, & Rieger, 2010). This should be examined in future studies, in which a priori predictions of this type can be made. Regardless, it is important to note that these results do not conflict with the inclusion of sex as a moderating variable in the integrative mediation framework (Hatzenbuehler, 2009). Hatzenbuehler posited that sex would moderate some, not all, paths and did not predict a universal direction for these effects.

Another notable discrepancy between the study’s Hypotheses (5 and 6) and results is that the direct path from active concealment to rumination was insignificant when controlling for expectations of rejection and self-stigma, resulting in active concealment being excluded from the final model. This is in contrast to an intermediary model in which the other proximal stressor variables were not included and the path from active concealment to psychological distress was significant and positive as predicted. However, this finding is in line with those of Velez et al. (2013), who found a positive path from avoiding references to sexual orientation in the workplace to distress, but no path from presenting a false heterosexual identity. The active concealment measure used here primarily tapped into one’s presentation and mannerisms, and so may be more like presenting a false heterosexual identity, which may be innocuous compared to this avoidance strategy and other proximal stressors. Another possible explanation is that there are indirect effects of active concealment on distress via these other proximal stressors. However, it is equally possible that there is, in fact, no effect of active concealment on distress, and the originally found relationship was merely due to high levels of self-stigma and expectations of rejection coinciding with higher levels of active concealment. Prospective studies will be required to determine which of these explanations is true.

Although all tested indirect effects were significant, many direct paths remained, indicating that most of the indirect relationships did not fully account for the respective direct relationships. Thus, while minority stress in sexual minority individuals does seem to operate via the above-outlined mechanisms, there may also be direct effects or indirect effects via variables not tested in the present study. Notably, there were relatively large direct paths from prejudice events to rumination and psychological distress. This suggests that interventions targeting the earlier intermediary variables identified in the current model (self-stigma and expectations of rejection) would be insufficient to prevent or completely mitigate rumination and distress associated with the experience of prejudice events. Hatzenbuehler’s (2009) framework specifies a number of other intermediary processes that require investigation, such as other coping responses and interpersonal factors. The findings also highlight the importance of continuing preventative interventions targeting societal stigma (Bartoş, Berger, & Hegarty, 2014).

There are several limitations to the present study that must be considered when interpreting the findings. Firstly, the data were cross-sectional, which means that direct tests of causality and temporal direction were not possible. Thus, this study should be viewed as important preparatory analyses. These will hopefully motivate and focus future prospective studies which can test causal pathways. Secondly, as data were taken online, participants were self-selected, and so the sample may not be representative of LGB individuals from the general population, a common problem for research in this area (Meyer & Wilson, 2009). Indeed, bisexual individuals in the present study did not appear to have higher levels of distress compared to lesbian/gay individuals, which prevented the data from being used to directly test why this disparity exists. However, findings regarding mental health disparities between bisexual and gay/lesbian individuals have not been entirely consistent (Plöderl & Tremblay, 2015; Ross et al., 2018). Moreover, the present sample was well characterized in that sexual orientation was assessed using multiple measures.

A further issue exists regarding the chosen measures. Since bisexual people have appeared to have different experiences of minority stress to lesbian/gay individuals, the tools used to measure stressors may not tap into the same construct in both groups. Indeed, research in this area generally suffers from an underdevelopment of measurement tools. Future research should attempt to develop tools which can compare different sexual minority groups’ heterogenous experiences of minority stress.

Another potential issue is the fact that this research was not focused on individuals’ resident in a specific country or of a particular race/ethnicity. While this facilitated the collection of a large sample and many of the differences driven by differing political and social climates would be captured by our measures, the observed relationships may differ across cultural and structural variations in stigma. For example, the availability of legal recourse for prejudice events or level of establishment of a national LGB community could potentially weaken the relationship between prejudice events and distress. This possibility should be followed up in future research using multilevel modeling or another similar method.

Additionally, participants were not prevented from participating based on their language ability. Though participants with low English ability are unlikely to have participated, due to difficulties in reading the recruitment materials, only providing the survey in English may have biased the sample. Finally, given the exclusion of those with a discordant gender identity and sex assigned at birth or a non-traditional sexual orientation identity, it is unclear to what extent these results would apply to individuals who are transgender, intersex, asexual, or those who identify with the non-traditional sexual orientation categories increasingly reported in contemporary settings (e.g., pansexual, asexual, or queer).

Nevertheless, if replicated in prospective studies these results may have important implications for the amelioration of psychological distress in LGB individuals in general. The indirect path from expectations of rejection to psychological distress via rumination and lack of a direct relationship implies that interventions targeting rumination (e.g., Watkins et al., 2011) could mitigate distress associated with this stressor. Furthermore, interventions specifically developed to address self-stigma, for which there was a direct path to distress, may also be warranted (Puckett & Levitt, 2015). Future research should prioritize rumination, self-stigma, and expectations of rejection as potential mediators of the effects of CGN and minority stressors on distress, due the significant indirect effects identified in this study; however, as the remaining relationship between prejudice events and distress was of non-trivial size, others will need to be identified.

## Electronic supplementary material

Below is the link to the electronic supplementary material.

Supplementary material 1 (DOCX 29 kb)

## Footnotes

1 Strictly speaking, stressors are typically defined as external, not internal, events and conditions (e.g., Wheaton & Montazer, 2010). We use the term consistent with Meyer (2003) and the bulk of minority stress research.

2 While these groups were not included in the current study, the data were collected as part of a larger research project.
