# Supplementary material for: Minority Stressors, Rumination, and Psychological Distress in Lesbian, Gay, and Bisexual Individuals
Source: Arch Sex Behav. 2019 Jul 22;49(2):661–80. doi: 10.1007/s10508-019-01502-2 (PMC7031186; doi:10.1007/s10508-019-01502-2)
Supplement: Supplementary file 1 — Supplementary material 1 (DOCX 29 kb) [file 10508_2019_1502_MOESM1_ESM.docx]

# Minority Stressors, Rumination and Psychological Distress in Lesbian, Gay and Bisexual

# Individuals

# SUPPLEMENTAL MATERIALS

# New Questionnaires

# These measures are distributed under the terms of the Creative Commons Attribution-NonCommercial-ShareAlike 4.0 International (https://creativecommons.org/licenses/by-nc-sa/4.0/), which permits unrestricted non-commercial use, distribution, and reproduction in any medium, provided you give appropriate credit to the original author(s) and the source, provide a link to the Creative Commons license, and indicate if changes were made. If you transform or build upon the material, you must distribute your contributions under the same license as the original. Please send a copy of the report from your study to (information masked) if you use or adapt any of these scales.

# Table S1

# *Gender and Sexual Minority Microaggressions Scale*

| The following questions ask about recent experiences you’ve had. Please note that these are experiences that people can have whether they are LGBT+ or not. Please indicate how often you have experienced each in the past year. | | | | | |
| --- | --- | --- | --- | --- | --- |
|  | Never | Rarely | Sometimes | Often | All of the Time |
| 1. People assuming you are attracted to them or willing to have sex with them simply because you are LGBT+ or they perceive you to be LGBT+. | 1 | 2 | 3 | 4 | 5 |
| 2. People assuming that you match an LGBT+ stereotype (e.g. assuming that you are knowledgeable about interior design if a man or carpentry if a woman). | 1 | 2 | 3 | 4 | 5 |
| 3. People accusing you of being defensive or sensitive when talking about your gender identity or sexual orientation. | 1 | 2 | 3 | 4 | 5 |
| 4. People referring to you as a gender you do not identify as or using incorrect pronouns when referring to you. | 1 | 2 | 3 | 4 | 5 |
| 5. People expecting you to act like a gender you do not identify as (e.g. expecting you to act stereotypically male if you don't identify as a man or stereotypically female if you don't identify as a woman). | 1 | 2 | 3 | 4 | 5 |
| 6. People suggesting that your sexual orientation or gender identity is just a phase, a choice or not real. | 1 | 2 | 3 | 4 | 5 |
| 7. People changing the subject/topic when reference to your sexual orientation or gender identity comes up. | 1 | 2 | 3 | 4 | 5 |
| 8. Finding it hard to find public facilities such as washrooms or toilets that you feel safe or comfortable using because you are LGBT+ or perceived as LGBT+. | 1 | 2 | 3 | 4 | 5 |
| 9. People finding you fascinating or exotic because you are LGBT+ or they perceive you to be LGBT+. | 1 | 2 | 3 | 4 | 5 |
| 10. People telling you to stop acting stereotypically LGBT+ (e.g. telling you to stop acting gay, or feminine for men, or masculine for women). | 1 | 2 | 3 | 4 | 5 |
| 11. People asking you invasive questions because you are LGBT+ or they perceive you to be LGBT+. | 1 | 2 | 3 | 4 | 5 |
| 12. People using words relating to LGBT+ people when talking about something or someone bad that is not LGBT+ related (e.g. calling something annoying “gay”). | 1 | 2 | 3 | 4 | 5 |
| *Note.* Scores are calculated by computing the mean of all items. If you are only taking data on cisgender sexual minority individuals, items 4, 5 and 8 can be discarded. The remaining items form the Sexual Minority subscale. Higher scores indicate more frequent experiences of microaggressions for all versions. | | | | | |

# Table S2

# *Gender and Sexual Minority Presentation Management Inventory*

| Some people engage in strategies in order to not appear LGBT+. Please indicate whether these items apply to you, only counting times in which you do this to not appear LGBT+ (irrespective of whether you are or not). | | | | | |
| --- | --- | --- | --- | --- | --- |
|  | Never | Rarely | Sometimes | Often | All of the Time |
| 1. I try to control how I talk (e.g. the pitch of my voice). | 1 | 2 | 3 | 4 | 5 |
| 2. I try to modify my gestures and mannerisms. | 1 | 2 | 3 | 4 | 5 |
| 3. I try to act more masculine or feminine. | 1 | 2 | 3 | 4 | 5 |
| 4. I check myself in order to see if there is anything that gives me away. | 1 | 2 | 3 | 4 | 5 |
| 5. I try change my appearance. | 1 | 2 | 3 | 4 | 5 |
| *Note.* Scores are calculated by computing the mean of all items. Higher scores indicate more frequent attempts to conceal gender and/or sexual minority status. | | | | | |

# Table S3

# *Vigilance for Others’ Suspicions Scale*

| Please read each of the items below and indicate how often you do the following in each of these situations. | | | | | |
| --- | --- | --- | --- | --- | --- |
|  | Never | Rarely | Sometimes | Often | All of the Time |
| 1. I become preoccupied with whether people suspect me of being LGBT+. | 1 | 2 | 3 | 4 | 5 |
| 2. I pay close attention to whether people suspect me of being LGBT+. | 1 | 2 | 3 | 4 | 5 |
| 3. I am quick to notice changes in how someone is treating me if they have reason to suspect me of being LGBT+. | 1 | 2 | 3 | 4 | 5 |
| *Note.* Scores are calculated by computing the mean of all items. Higher scores indicate more frequent vigilance for suspicions of one’s gender and/or sexual minority status. | | | | | |

| **Exploratory Analyses**  Table S4  *Path from Childhood Gender Conformity to Prejudice Events* | | | | |
| --- | --- | --- | --- | --- |
|  | Bisexual Women | Bisexual Men | Lesbians | Gay Men |
| Unconstrained, standardised beta | .16^***^ | .18^***^ | .10^***^ | .28^***^ |
| χ^2^ when paths constrained to be equal | | | | |
| Bisexual Women | - |  |  |  |
| Bisexual Men | .69 | - |  |  |
| Lesbians | 3.95^*^ | 6.28^*^ | - |  |
| Gay Men | 5.46^*^ | .89 | 26.15^***^ | - |
| ^*^*p* < .05, ^***^*p* <.001. | | | | |
| Table A-2  *Path from Childhood Gender Conformity to Outnesss* | | | | |
|  | Bisexual Women | Bisexual Men | Lesbians | Gay Men |
| Unconstrained, standardised beta | .00 | .13^***^ | .09^**^ | .13^***^ |
| χ^2^ when paths constrained to be equal | | | | |
| Bisexual Women | - |  |  |  |
| Bisexual Men | 7.49^**^ | - |  |  |
| Lesbians | 3.17 | 2.58 | - |  |
| Gay Men | 9.80^**^ | .59 | 2.07 | - |
| ^**^*p* <.01 | | | | |
| Table S5  *Path from Outness to Prejudice Events* | | | | |
|  | Bisexual Women | Bisexual Men | Lesbians | Gay Men |
| Unconstrained, standardised beta | .26^***^ | .41^***^ | .13^***^ | .05 |
| χ^2^ when paths constrained to be equal | | | | |
| Bisexual Women | - |  |  |  |
| Bisexual Men | 3.64 | - |  |  |
| Lesbians | 8.57^**^ | 22.42^***^ | - |  |
| Gay Men | 26.17^***^ | 49.71^***^ | 3.05 | - |
| ^*^*p* < .05, ^**^*p* <.01, ^***^*p* <.001. | | | | |
| Table S6  *Path from Outness to Self-Stigma* | | | | |
|  | Bisexual Women | Bisexual Men | Lesbians | Gay Men |
| Unconstrained, standardised beta | -.26^***^ | -.31^***^ | -.42^***^ | -.37^***^ |
| χ^2^ when paths constrained to be equal | | | | |
| Bisexual Women | - |  |  |  |
| Bisexual Men | 1.19 | - |  |  |
| Lesbians | 19.95^***^ | 6.59^**^ | - |  |
| Gay Men | 19.02^***^ | 4.96^*^ | .49 | - |
| ^*^*p* < .05, ^**^*p* <.01, ^***^*p* <.001. | | | | |

| Table S7  *Path from Outness to Expectations of Rejection* | | | | |
| --- | --- | --- | --- | --- |
|  | Bisexual Women | Bisexual Men | Lesbians | Gay Men |
| Unconstrained, standardised beta | -.25^***^ | -.36^***^ | -.47^***^ | -.46^***^ |
| χ^2^ when paths constrained to be equal | | | | |
| Bisexual Women | - |  |  |  |
| Bisexual Men | 3.87^*^ | - |  |  |
| Lesbians | 27.10^***^ | 6.31^*^ | - |  |
| Gay Men | 34.54^***^ | 7.13^**^ | .02 | - |
| ^*^*p* < .05, ^**^*p* <.01, ^***^*p* <.001. | | | | |
| Table S8  *Path from Outness to Psychological Distress* | | | | |
|  | Bisexual Women | Bisexual Men | Lesbians | Gay Men |
| Unconstrained, standardised beta | -.08^*^ | -.04 | -.09^**^ | -.03 |
| χ^2^ when paths constrained to be equal | | | | |
| Bisexual Women | - |  |  |  |
| Bisexual Men | .58 | - |  |  |
| Lesbians | .32 | 1.45 | - |  |
| Gay Men | 1.04 | .00 | 2.40 | - |
| ^*^*p* < .05, ^**^*p* <.01. | | | | |

| Table S9  *Path from Prejudice Events to Self-Stigma* | | | | |
| --- | --- | --- | --- | --- |
|  | Bisexual Women | Bisexual Men | Lesbians | Gay Men |
| Unconstrained, standardised beta | .23^***^ | .29^***^ | .23^***^ | .25^***^ |
| χ^2^ when paths constrained to be equal | | | | |
| Bisexual Women | - |  |  |  |
| Bisexual Men | 2.60 | - |  |  |
| Lesbians | .66 | .78 | - |  |
| Gay Men | 3.67 | .06 | .77 | - |
| ^***^*p* <.001. | | | | |
| Table S10  *Path from Prejudice Events to Expectations of Rejection* | | | | |
|  | Bisexual Women | Bisexual Men | Lesbians | Gay Men |
| Unconstrained, standardised beta | .69^***^ | .73^***^ | .52^***^ | .59^***^ |
| χ^2^ when paths constrained to be equal | | | | |
| Bisexual Women | - |  |  |  |
| Bisexual Men | 2.28 | - |  |  |
| Lesbians | 1.71 | 6.44^*^ | - |  |
| Gay Men | .05 | 3.19 | 1.51 | - |
| ^*^*p* < .05, ^***^*p* <.001. | | | | |

| Table S11  *Path from Prejudice Events to Rumination* | | | | |
| --- | --- | --- | --- | --- |
|  | Bisexual Women | Bisexual Men | Lesbians | Gay Men |
| Unconstrained, standardised beta | .10 | .04 | .26^***^ | .23^***^ |
| χ^2^ when paths constrained to be equal | | | | |
| Bisexual Women | - |  |  |  |
| Bisexual Men | .49 | - |  |  |
| Lesbians | 7.96^**^ | 11.52^***^ | - |  |
| Gay Men | 8.34^**^ | 12.21^***^ | .07 | - |
| ^**^*p* <.01, ^***^*p* <.001. | | | | |
| Table S12  *Path from Prejudice Events to Psychological Distress* | | | | |
|  | Bisexual Women | Bisexual Men | Lesbians | Gay Men |
| Unconstrained, standardised beta | .25^***^ | .24^***^ | .27^***^ | .23^***^ |
| χ^2^ when paths constrained to be equal | | | | |
| Bisexual Women | - |  |  |  |
| Bisexual Men | .06 | - |  |  |
| Lesbians | 2.02 | .93 | - |  |
| Gay Men | .62 | .13 | .64 | - |
| ^***^*p* <.001. | | | | |

| Table S13  *Path from Self-Stigma to Rumination* | | | | |
| --- | --- | --- | --- | --- |
|  | Bisexual Women | Bisexual Men | Lesbians | Gay Men |
| Unconstrained, standardised beta | .10^**^ | .08 | .07 | .06^*^ |
| χ^2^ when paths constrained to be equal | | | | |
| Bisexual Women | - |  |  |  |
| Bisexual Men | .47 | - |  |  |
| Lesbians | .48 | .00 | - |  |
| Gay Men | 1.04 | .02 | .05 | - |
| ^*^*p* < .05, ^**^*p* <.01. | | | | |
| Table S14  *Path from Self-Stigma to Psychological Distress* | | | | |
|  | Bisexual Women | Bisexual Men | Lesbians | Gay Men |
| Unconstrained, standardised beta | .05 | .08^*^ | .05 | .12^***^ |
| χ^2^ when paths constrained to be equal | | | | |
| Bisexual Women | - |  |  |  |
| Bisexual Men | .56 | - |  |  |
| Lesbians | .04 | .31 | - |  |
| Gay Men | 3.93^*^ | .90 | 2.90 | - |
| ^*^*p* < .05, ^***^*p* <.001. | | | | |

| Table S15  *Path from Expectations of Rejection to Rumination* | | | | |
| --- | --- | --- | --- | --- |
|  | Bisexual Women | Bisexual Men | Lesbians | Gay Men |
| Unconstrained, standardised beta | .27^***^ | .43^***^ | .25^***^ | .36^***^ |
| χ^2^ when paths constrained to be equal | | | | |
| Bisexual Women | - |  |  |  |
| Bisexual Men | 2.62 | - |  |  |
| Lesbians | .12 | 4.55^*^ | - |  |
| Gay Men | 2.43 | .13 | 4.97^*^ | - |
| ^*^*p* < .05, ^***^*p* <.001. | | | | |
| Table S16  *Path from Rumination to Psychological Distress* | | | | |
|  | Bisexual Women | Bisexual Men | Lesbians | Gay Men |
| Unconstrained, standardised beta | .56^***^ | .56^***^ | .55^***^ | .53^***^ |
| χ^2^ when paths constrained to be equal | | | | |
| Bisexual Women | - |  |  |  |
| Bisexual Men | .57 | - |  |  |
| Lesbians | .43 | .03 | - |  |
| Gay Men | .08 | 1.14 | 1.04 | - |
| ^***^*p* <.001. | | | | |
